# Recent advances in radiotracers targeting norepinephrine transporter: structural development and radiolabeling improvements

**DOI:** 10.1007/s00702-020-02180-4

**Published:** 2020-04-09

**Authors:** Xinyu Chen, Takashi Kudo, Constantin Lapa, Andreas Buck, Takahiro Higuchi

**Affiliations:** 1 Department of Nuclear Medicine, University Hospital of Würzburg, Oberdürrbacher Str. 6, 97080 Würzburg, Germany; 2 Comprehensive Heart Failure Center, University Hospital of Würzburg, Würzburg, Germany; 3 Department of Nuclear Medicine, University Hospital of Augsburg, Augsburg, Germany; 4 Department of Radioisotope Medicine, Atomic Bomb Disease Institute, Nagasaki University, Nagasaki, Japan; 5 Graduate School of Medicine, Dentistry and Pharmaceutical Sciences, Okayama University, Okayama, Japan

**Keywords:** Norepinephrine transporter, Benzylguanidine, Phenethylguanidine, Antidepressant, Organic cation transporter

## Abstract

The norepinephrine transporter (NET) is a major target for the evaluation of the cardiac sympathetic nerve system in patients with heart failure and Parkinson's disease. It is also used in the therapeutic applications against certain types of neuroendocrine tumors, as exemplified by the clinically used ^123/131^I-MIBG as theranostic single-photon emission computed tomography (SPECT) agent. With the development of more advanced positron emission tomography (PET) technology, more radiotracers targeting NET have been reported, with superior temporal and spatial resolutions, along with the possibility of functional and kinetic analysis. More recently, fluorine-18-labelled NET tracers have drawn increasing attentions from researchers, due to their longer radiological half-life relative to carbon-11 (110 min vs. 20 min), reduced dependence on on-site cyclotrons, and flexibility in the design of novel tracer structures. In the heart, certain NET tracers provide integral diagnostic information on sympathetic innervation and the nerve status. In the central nervous system, such radiotracers can reveal NET distribution and density in pathological conditions. Most radiotracers targeting cardiac NET-function for the cardiac application consistent of derivatives of either norepinephrine or MIBG with its benzylguanidine core structure, e.g. ^11^C-HED and ^18^F-LMI1195. In contrast, all NET tracers used in central nervous system applications are derived from clinically used antidepressants. Lastly, possible applications of NET as selective tracers over organic cation transporters (OCTs) in the kidneys and other organs controlled by sympathetic nervous system will also be discussed.

**Electronic supplementary material:**

The online version of this article (10.1007/s00702-020-02180-4) contains supplementary material, which is available to authorized users.

## Introduction

**Fig. 1:**
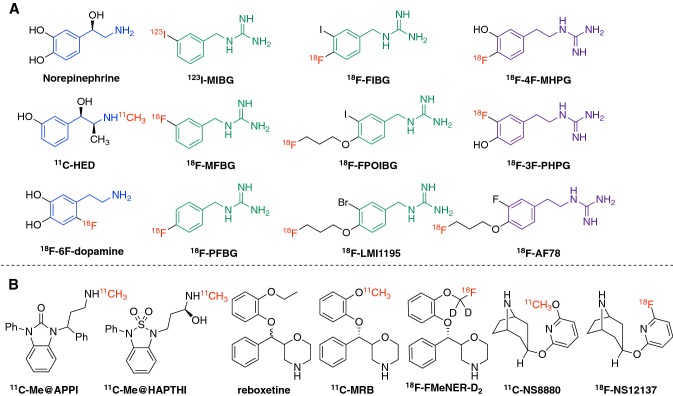
Currently reported radiotracers targeting NET. **a** Norepinephrine and NET tracers for cardiac imaging. From a structural point of view, the blue moiety represents the common core structure norfenefrine, dark green represents benzylguanidine core structure, and purple represents phenethylguanidine moiety. Red symbolizes radioactive isotopes (Chen et al. 2015, 2019). **b** The CNS NET tracers derived from antidepressants. This figure was created using ChemDraw 16 for Mac

The norepinephrine transporter (NET) serves as the main mechanism for terminating noradrenergic signaling at the synaptic gap and is essential for the reuptake of the neurotransmitter norepinephrine (NE), as well as the extraction of radiotracers targeting NET both in the central nervous system (CNS) and peripheral sympathetic nervous system (SNS). First, it is one of the main targets for pharmacological intervention of related diseases, especially in the CNS. Drugs targeting NET are therapeutically used in the treatment of disorders including depression, attention-deficit hyperactivity disorder (ADHD), and feeding disturbances (Bönisch and Brüss 2006). Second, in common cardiac diseases, such as congestive heart failure (CHF) and ischemia, the cardiac SNS appears to be malfunctioning (Schroeder and Jordan 2012). Sympathetic innervation and function, as represented by the uptake kinetics of NET tracers, is used as a parameter for the diagnosis of heart diseases. Third, in Parkinson’s disease (PD), there is growing evidence that, besides depletion of dopamine, additional loss of noradrenergic neurons could also be involved in the clinical expression of motor symptoms (Delaville et al. 2011). Thus, NET is used as a marker to reveal the neurodegeneration and to diagnose parkinsonism. Lastly, due to the specific overexpression/up-regulation of NET in certain neuroendocrine tumors, radiolabeled compounds could also be used for chemotherapy, as seen with the use of currently the only clinically utilized NET ligand, ^123/131^I-*meta*-iodobenzylguanidine (^123/131^I-MIBG), against neuroendocrine tumors. Physiologically, the extracted NEs are stably stored in the storage vesicles within the presynaptic neurons, shielding them from metabolism by monoamine oxidase (MAO). This mechanism is crucial for the diagnostic applications, as it reflects the systemic distribution and/or regional expression of NET in pathophysiological conditions. The current review focuses on the introduction of recent developments in NET-targeting radiotracers for both cardiac and CNS diagnostic applications using either single-photon emission computed tomography (SPECT) or positron emission tomography (PET) technology. All the chemical structures of the radiotracers listed in this review are illustrated in Fig. 1, with a particular emphasis on advantageous fluorine-18-labeled ones. Radiotracers targeting cardiac NET are mainly developed from benzylguanidine or metaraminol, which are metabolically stable against MAO, while radiotracers for CNS imaging are derivatives of known NET-selective antidepressants. The discussion will not only examine their potential applications in HF, tumor diagnosis, and CNS disorders, but also, more importantly, analyze new tracer design from a chemical and structural perspective, addressing the specificities of different transporters along with the pros and cons of the radiolabeling procedure. NET is the reuptake transporter of neurotransmitters or radiotracers into the neuronal terminals. Therefore, the term “uptake-1” is used in publications (including the present one) to represent NET-mediated tracer uptake. Traditionally, any extra-neuronal uptake would be labeled “uptake-2”, the molecular mechanism of which is not clearly identified, but will be explored in details in the discussion section.

### SPECT tracers targeting NET

#### ^123^I-MIBG

As the only NET-targeting radiotracer currently used clinically, ^123^I-MIBG, using SPECT technology, has demonstrated favorable results in the diagnosis of cardiac diseases and has confirmed the utility of independent risk stratification in adrenergic imaging. These conclusions derive from the accumulation of several years of clinical trials data in the landmark “ADreview Myocardial Imaging for Risk Evaluation in Heart Failure” (ADMIRE-HF) study, as well as the follow-up ADMIRE-HF extra study (Travin 2017). Moreover, ^123^I-MIBG is used as a risk stratification tool for potential implantation of cardioverter defibrillators (ICD) in HF patients. The currently recruiting event-driven ADMIRE-ICD trial will provide further insight into its efficacy in patient management and determine which patients benefits the most from the implantation (Travin 2017). In addition, a number of malignancies of endocrine origin also feature high NET expression, along with monoamines uptake and storage, which forms the foundation for using ^131^I-MIBG as a therapeutic agent against neuroblastoma and various neuroendocrine tumors (Inaki et al. 2017; Agrawal et al. 2018). Pandit-Taskar and Modak (2017) have presented an overview of NET as a target for theranostics, with a particular focus on the current role and broader application of MIBG and its analogues in neuroendocrine tumors. Very recently, based on the clinical study MIP-IB12B (NCT00874614), the Food and Drug Administration of the US has approved ^131^I-MIBG (AZEDRA, Progenics Pharmaceuticals, Inc.) for the anticancer therapy of adult and pediatric patients (12 years and older) with pheochromocytoma (PCC) or paraganglioma (PGL) (Pryma et al. 2019). Now, it is on an expanded study to evaluate the safety and tolerability (NCT02961941). In addition, due to the interaction between the sympathetic components of the various organs controlled by autonomic nervous system (e.g. kidney, brain, and periphery), the application of ^123^I-MIBG and analogous NET radiotracers can also be broadened to assess the pathophysiological conditions in extra-cardiac organs, such as renal denervation and PD (Travin 2019). In the last couple of years, MIBG scintigraphy has been considered for use in the diagnosis of early stage parkinsonian disorders. A study involving 600 patients has demonstrated that ^123^I-MIBG has the diagnostic ability to distinguish PD from atypical parkinsonism. Almost half of the patients displayed positive correlation between decreased tracer uptake and PD. These findings demonstrated its sufficient diagnostic accuracy for detecting the early phase of PD (Kawazoe et al. 2019). When diagnosing PD, use of ^123^I-ioflupane SPECT with ^123^I-MIBG cardiac scintigraphy association, has made it possible to achieve the most appropriate diagnosis in over 90% cases (Niimi et al. 2017; Nuvoli et al. 2018; Okada et al. 2018). In addition, ^123^I-MIBG is also helpful for differentiating Alzheimer’s disease (AD) from Lewy Body dementias (Nuvoli et al. 2018). Most recently, a study involving 115 patients indicated a potential role for immediate (5 min) or early (15 min) acquisition (replacing that of 240 min) in PD patients, which paves the way to a faster validation (Frantellizzi et al. 2020).

^123^I-MIBG is the first in the benzylguanidine series of NET tracers with guanidine moieties. It features a chemically polar structure, which ensures its long-term storage in granular vesicles after extraction. Its biological stability against MAO has set up a paradigm for subsequent tracer design. Despite being introduced 30 years ago, its disadvantages as a SPECT radiotracer limit further application. Among these disadvantages are poorer spatial resolution compared to PET, limited sensitivity in small lesions/small sized tumors, the necessity of several scanning protocols, as well as a delay between the tracer administration and result obtainment (Pfluger and Piccardo 2017). Therefore, in the last two decades, PET, rather than SPECT tracers has become the focus of research.

### PET tracers targeting NET

#### ^11^C-HED

^11^C-Hydroxyephedrine (HED), as one of the first PET tracers targeting NET, has been investigated intensively in Japan and the US, with studies involving a variety of patients confirming its feasibility in quantifying not only regional sympathetic denervation, but also other conditions as well (Fujita et al. 2016; Hiroshima et al. 2017; Aikawa et al. 2017; Wang et al. 2018). Studies by Fujita et al. on ^11^C-HED PET involving 60 patients with left ventricular (LV) dysfunction as a predictor of all-cause death. Thirteen deceased patients were associated with significantly lower HED retention than those who survived (7.1 vs. 9.0, *p* = 0.015). This result demonstrated that cardiac sympathetic dysfunction as measured by ^11^C-HED PET (0.762 as retention per unit (/min) as a hazard ratio) is related to poor survival rate in patients with LV dysfunction, independent of age (Fujita et al. 2016). This is in accordance with prior systematic investigation performed by only two other research groups (Pietilä et al. 2001; Fallavollita et al. 2014). Furthermore, by enrolling ten controls and thirteen patients, it was possible to correlate the ^11^C-HED PET with myocardial blood flow. The correlation coefficient demonstrated a significantly high relationship for both the whole left ventricle and three coronary territories, illustrating the potential of ^11^C-HED for simultaneous assessment of perfusion and neuronal function within a single scan, for the determination of mismatch (Hiroshima et al. 2017). In addition to global assessment, ^11^C-HED has also been used for investigating regional sympathetic denervation in HF patients with preserved left ventricular ejection fraction (HFpEF). The results obtained from 34 patients, along with 11 age-matched volunteers without HF, showed the global ^11^C-HED retention index to be significantly lower and more heterogeneous in HFpEF patients than in volunteers (*p* < 0.01). Regional sympathetic denervation is associated with both contractile dysfunction and the extent of myocardial scarring in patients with HFpEF (*p* ≤ 0.001). It is suggested that regional sympathetic denervation may provide an integrated measure of myocardial damage in HFpEF patients (Aikawa et al. 2017). One of the advantages of cardiac imaging using PET tracers is the possibility of performing quantification analysis. Therefore, in an effort to improve and expand the use of kinetic modeling, Wang et al., estimated the myocardiac tissue volume of distribution (*V*_T_) using both Logan and multilinear analysis 1 (MA1) graphical methods, while comparing with the one-tissue-compartment (1TC) standard model values. Both the MA1 and Logan models exhibited good agreement with 1TC, with similar regional patterns and global median values, as well as good-to-excellent test–retest repeatability (Wang et al. 2018). The Logan model underestimated *V*_T_ due to the recognized noise bias. Logan and MA1 both exhibited similar test–retest variability, suggesting that they may be used in addition to 1TC in the modeling of ^11^C-HED kinetics, with benefits of greater computational simplicity and the ability to mathematically visualize kinetic parameters for better quality assurance (Wang et al. 2018). Wu et al., investigated the reproducibility and repeatability of ^11^C-HED quantification of global cardiac innervation, regional denervation and myocardial blood flow (MBF) in 20 patients. Their results demonstrated a more reliable repeatability with the simple retention index (RI) method as opposed to that of the complex *V*_T_ in quantitative measurement of both global and regional innervation, supporting the use of RI over *V*_T_ for clinical trials in prediction of cause-specific mortality from sudden cardiac arrest (Wu et al. 2019). Most recently, ^11^C-HED has also been used for the evaluation of adaptive servo-ventilation (ASV) in improving cardiac function of HF patients while comparing to ^123^I-MIBG. Increased heart/mediastinum ratio of ^123^I-MIBG imaging along with increased global ^11^C-HED RI confirmed the feasibility of ASV by demonstrating improved sympathetic nerve function (Tokuda et al. 2019). In addition to its clinical application, researchers continue to use ^11^C-HED as a preclinical tool for basic research studies, including in a rat model with transient myocardial ischemia. A large defect of ^11^C-HED due to the denervation in ischemic areas has confirmed the increased susceptibility of sympathetic neurons over cardiomyocytes during ischemic injury. It has been shown that the ^11^C-HED uptake defect areas in both the subacute and chronic phases after transient ischemia were clearly larger than the perfusion defect areas in the midventricular wall. Partial reinnervation could be observed as the uptake of ^11^C-HED recovered in the subepicardial portion in the chronic phase (Werner et al. 2016). Furthermore, the potential cold mass effect of ^11^C-HED (specific activities 0.2–141.9 GBq/µmol) in healthy Wistar rats (ranging 0.2–60.4 µg/kg cold mass) was also investigated, in which a dose-dependent reduction of cardiac ^11^C-HED uptake with different specific activities was observed. With the cold mass dose increased from 0.2, 1, 10 to 34 µg/kg, a subsequent decrease of tracer uptake in the left ventricular myocardium could be clearly recorded. Time–activity curves demonstrated that at the highest cold dose (34 µg/kg), the washout increased considerably, while both the low-dose curves (0.2 and 1 µg/kg) remained relatively stable (Werner et al. 2018). In addition, in a longitudinal setting, the cardiac uptake of ^11^C-HED in rat hearts reduced from M5, M11 to M15, while ^18^F-Fludeoxyglucose (^18^F-FDG) uptake remained stable during the same time period, indicating preserved myocardial viability. As has been previously reported, when compared to ^123^I-MIBG, the cardiac uptake of ^11^C-HED is at equilibrium and relies on the continuous leaking–reuptaking pathway by NET (Werner et al. 2015). Accordingly, an age-related deterioration of NET functionality might explain these findings, which are in accordance with results reported in human studies using other NET tracers (Tsuchimochi et al. 1995; Li et al. 2003).

As a radiotracer targeting NET, the applications of ^11^C-HED are not only limited to the cardiac conditions such as HF. Wong et al. (2017) investigated the regional patterns of cardiac sympathetic denervation in PD using ^11^C-HED PET and determined the denervation rate of 39 patients. Their findings revealed the heterogeneity of cardiac sympathetic denervation in PD. Over a 2-year interval, progressive decline occurred at a modest rate (as observed by ^11^C-HED PET scans), with a regional preferential pattern, consistent with ^18^F-6-fluorodopamine results. In addition, similar to the application of ^123^I-MIBG in the diagnosis of neuroendocrine tumors, ^11^C-HED could be used as an accurate tool for the diagnosis/ruling out of PCC and PGL in complex clinical scenarios, in contrast to CT/MRI characterization. In a 2019 study by Vyakaranam et al. (2019), ^11^C-HED PET/CT was performed for a cohort of 102 patients, where 19 patients were correctly identified as having PCC, 6 with PGL, and 75 successfully excluded from having either. Moreover, using ^11^C-HED PET as an indicator of sympathetic innervation (expressed as RI%), it is possible to predict levels of supraclavicular brown adipose tissue (BAT) in lean normal adult subjects. Furthermore, it has been seen that cold-induced decrease in white adipose tissue (WAT) energy expenditure is less pronounced in subjects with high amounts of activated BAT (Muzik et al. 2017).

From structural point of view, ^11^C-HED is derived from the earlier reported metaraminol, which is biologically stable against MAO due to its ephedrine structure, where the *α*- and *N*-methyl groups are able to prevent/slow down the possible enzymatic oxidation/deamination. ^11^C-HED contains two chiral centers. Although both stereoisomers have demonstrated high initial accumulation in murine model hearts, the (+)-isomer features a much faster efflux compared to the (−)-isomer, with effluxes of *T*_1/2_ 2–3 h vs. 2–3 days, respectively (Van Dort et al. 2000). Therefore, in potential kinetic studies, the (−)-stereoisomer is always used. In addition, due to its specific ephedrine structure, which is dissimilar from the other radiotracers possessing a guanidine moiety, its in vivo storage and release kinetics differ, as has been mentioned above (Werner et al. 2015).

#### Advantages of fluorine-18 over carbon-11-labeled PET radiotracers

Over the last two decades, increasing attention has been placed on fluorine-18-labelled PET tracers (whose radioactive half-life is 110 min) due to the following advantages over carbon-11-labelled tracers (whose half-life is 20 min): (1) it is more cost effective, given the possibility for its distribution from a central cyclotron facility, reducing the requirement of a costly on-site cyclotron; (2) there exists the potential of delayed and prolonged scans due to its longer half-life; (3) spatial resolution in PET imaging is improved as a consequence of its shorter travel distance prior to annihilation, a result of the low positron energy of fluorine-18; (4) it allows for further alternatives and flexibility in novel tracer design, along with the possibility of improving the tracer stability against metabolism at sensitive positions (Kobayashi et al. 2017). As a result, the designing of new NET tracers will focus on the use of fluorine-18 for subsequent development as to take the full advantages of PET imaging technology (Fig. 1a).

#### ^***18***^***F-6F-dopamine***

^18^F-6F-dopamine is the metabolite of ^18^F-6F-DOPA and can be transported by NET into cardiac sympathetic nerves. The dosimetry of ^18^F-6F-dopamine for clinical PET scan has been estimated based on the results from rats and dogs (Goldstein et al. 1991). In initial human study, ^18^F-6F-dopamine is fast extracted by the sympathetic nerves, where it is translocated into vesicles (Deep et al. 1997). It can provide the neuronal uptake and the turnover of vesicles in the human heart (Goldstein et al. 1993). However, due to its primary amine structure, it undergoes fast metabolism in vivo with only 1–2% as parent tracer after 10 min injection (Ding et al. 1993). To visualize the cardiac sympathetic neurons, high dose will be needed due to the large excretion from urinary collecting system (Goldstein et al. 1994). After the administration of ^18^F-6F-dopamine, the concentration of the tracer in the heart declined bi-exponentially from the peak value. Tyramine chase (injection after radiotracer administration) could increase the speed of tracer washout (Goldstein et al. 1997). Using ^18^F-6F-dopamine PET, it is possible to prove the decreased SNS function in hypertrophic cardiomyopathy patients (Li et al. 2000). Goldstein and coworkers have further developed a kinetic model for ^18^F-6F-dopamine, which can successfully predict the tracer kinetics in vivo under certain pharmacological manipulations (Goldstein et al. 2002).

Similar to MIBG, due to its ability to be taken up by NET, ^18^F-6F-dopamine is also used for diagnostic localization of PCC with high sensitivity (Pacak et al. 2001), with superior outcome compared to ^123^I-MIBG (Ilias et al. 2003; Mamede et al. 2006; Kaji et al. 2007). In several patient studies, ^18^F-6F-dopamine showed equal sensitivity to localize non-metastatic PCC when compared to ^123^I-MIBG, but much better for metastatic ones (Ilias et al. 2008; Timmers et al. 2009a, b). It is helpful to distinguish between physiological adrenal gland uptake and pathological PCC uptake (Timmers et al. 2007). In patient study with mostly adrenal PGL, ^18^F-6F-dopamine is recommended over ^18^F-FDG, ^123^I-MIBG and ^18^F-DOPA (Timmers et al. 2009a, b), whereas when detecting head and neck PGL, ^18^F-DOPA proved to be the most effective one (King et al. 2011). Other application of ^18^F-6F-dopamine include imaging proof of denervation in the painful feet of patients with diabetic neuropathy (Tack et al. 2002), detection of medullary thyroid cancer (Gourgiotis et al. 2003), ageing-related cardiac SNS changes (Li et al. 2003), cardiac and extra-cardiac sympathetic denervation in PD with orthostatic hypotension patients (Tipre and Goldstein 2005), hypo-innervation in familial dysautonomia (Goldstein et al. 2008), prediction of PD in at-risk individuals (Goldstein et al. 2018). ^18^F-6F-dopamine was initially radiolabeled using electrophilic labeling method ^18^F-F_2_ with only 2.6% radiochemical yield and 13.2 GBq/µmol specific acitivity (Eskola et al. 2012). After changing to a nickel-mediated radiofluorination method, the yield was improved to 12% (Zlatopolskiey et al. 2015). Most recently, the use of iodonium salt as precursor in a one-pot two-step automatic synthesis could increase the yield to 26% (Vāvere et al. 2018) that allows the clinical trial of pediatric neuroblastoma imaging (IND 138638).

#### ^***18***^***F-MFBG/PFBG***

To investigate the fluorine-18 derivatives of MIBG, two methods have been used to introduce the fluorine directly onto the benzylguanidine core structure: (1) replacing the iodine with fluorine directly to obtain ^18^F-*meta*- and *para*-fluorobenzylguanidine (^18^F-MFBG/PFBG, Garg et al. 1994); (2) adding an additional fluorine to the MIBG structure to get ^18^F-(4-fluoro-3-iodobenzyl)guanidine (^18^F-FIBG, Vaidyanathan et al. 1994, 1995). ^18^F-MFBG and PFBG were originally radiolabeled using a 3-step synthetic scheme. Selective uptake of both ^18^F-MFBG/PFBG isomers has been observed in mice at similar levels: slightly higher levels of adrenal uptake than in the heart, while the radiochemical yield of PFBG is relatively higher than MFBG (Garg et al. 1994). The specific uptake of ^18^F-MFBG and PFBG were similar when examined in rat C6 glioma cells stably transfected with the human NET (C6-hNET), but fourfold less than MIBG. PET imaging of ^18^F-MFBG has demonstrated comparable tumor accumulation to the SPECT tracer ^123^I-MIBG, but MFBG showed a significantly more rapid clearance (Zhang et al. 2014a). A follow-up study using five neuroblastoma cell lines and two xenografts expressing different levels of NET confirmed the lower affinity for NET and lower cellular uptake of MFBG than MIBG, but the in vivo imaging and tissue radioactivity concentration measurements still demonstrated higher MFBG xenograft uptake and tumor-to-normal organ ratios (Zhang et al. 2014b). Due to the development of iodonium salt as a precursor for the electron-rich benzene ring radiofluorination method, the radiolabeling of ^18^F-MFBG has been optimized to make the last step radiofluorination, followed by the total deprotection of Boc groups from guanidine moiety with an overall radiolabeling time less than 1 hour. The average yield was greatly improved from 10–15% to 31% with > 99% radiochemical purity, which permits the automated production of multi-dose batches of clinical grade MFBG (Hu et al. 2015). Based on these thorough preclinical evaluations, the first-in-human study was performed in ten patients, which showed excellent in vivo stability and safety as well as a favorable biodistribution with good targeting of lesions that makes it feasible for same-day imaging of neuroendocrine tumors. The maximum standardized uptake value (SUV_max_) of lesions was 8.6 ± 9.6 at 1–2 h and 9.2 ± 11.4 at 3–4 h after injection. It showed a two phases clearance from the blood pool and was primarily excreted from the kidneys. Due to this, the bladder was exposed to the highest and longest amount of radiation exposure faster than ^123^I-MIBG, counting 61–95% *vs* 11–26%, respectively, by 3 h after injection. ^18^F-MFBG demonstrated high promise for imaging in patients, especially for children with neuroblastoma (Pandit-Taskar et al. 2018).

#### ^***18***^***F-FIBG and analogues***

Analogously, Vaidyanathan et al., devised another method for the introduction of the extra fluorine-18 to MIBG, as opposed to replacing iodine, to form ^18^F-FIBG. Similar to the other fluorobenzylguanidine tracers, the original radiolabeling procedure consisted of multiple steps with a long synthesis time (130 min) and low radiochemical yield (5% decay corrected) due to the requirement of the electron-withdrawing group cyanide for the successful electrophilic radiofluorination. However, both in vitro binding studies on SK-N-SH human neuroblastoma cells and in vivo tissue distribution studies indicate comparable properties to MIBG (Vaidyanathan et al. 1994). Further in vitro assays of tracer binding were carried out to confirm the NET-specific and energy-dependent uptake mechanisms similar to those of MIBG. A blocking study using desipramine (DMI) in mice has further confirmed the NET specificity by reducing cardiac and adrenal uptake to 57% and 60%, respectively. Radiation dosimetry calculations also suggested that, as an important advantage, higher doses of ^18^F-FIBG could be administered to patients than ^124^I-MIBG (Vaidyanathan et al. 1995). Presumably due to the encouraging results obtained from ^18^F-FIBG, the same research group also reversely examined ^131^I-FIBG and the corresponding alpha-emitter analogue ^211^At-(3-astato-4-fluorobenzyl)guanidine (^211^At-AFBG), both of which were synthesized from the same precursor using similar radiolabeling methods with 50–60% and 65–70% yield, respectively (Vaidyanathan et al. 1996). Naturally, both ^131^I-FIBG and ^211^At-AFBG demonstrated characteristics comparable to those of their parent compound MIBG in terms of NET-specific uptake in both cell-based and mouse studies. It is noteworthy that both new analogues show higher stability against dehalogenation in mice when compared to ^131^I-MIBG or ^211^At-MABG. They particularly feature lower thyroidal uptake, presumably due to the introduction of fluorine—the strong electron-withdrawing effect of which stabilizes the C–I and C–At bond (Vaidyanathan et al. 1997a, b). Further cell studies suggested that when ^125^I-MIBG and ^125^I-FIBG metabolism occurs, it leads to the same metabolites, but not as the original halobenzylguanidine (Vaidyanathan et al. 2004). As previously reported, one of the main metabolites of MIBG is 4-hydroxy-3-iodobenzylguanidine (HIBG) (Mangner et al. 1986). As a consequence, the 4-fluoro substituent of FIBG greatly slows down the metabolism (i.e. defluorination and oxidation) when compared to MIBG. In a SK-N-SH cell study, 70% of radioiodine activity remained from ^125^I-FIBG after 3 days of experiment, compared to just 30% for ^125^I-MIBG. This offers a positive hint toward future tracer design: the introduction of a fluorine may provide not only the possibility of deriving the known tracer into an ^18^F-labeled PET tracer without major changes in characteristics, but may also introduce further benefits, such as improving the tracer stability in vivo by preventing the enzymatic degradation. Presumably due to their difficult radiolabeling procedure and low radiochemical yield, these tracers have not drawn much attention from researchers. Most recently, however, again benefiting from the development of iodonium salt as the precursor for electron-rich aromatic radiofluorination, Yamaguchi et al., optimized the radiolabeling of both ^18^F-FIBG and ^131^I-MIBG using a copper-mediated 2-step procedure with a mesityl(aryl)iodonium salt precursor. Their results reconfirm the feasibility of these compounds as diagnostic and therapeutic agents with high and specific accumulation in the PC12 xenograft tumor cells, along with high tumor-to-background ratios in mice studies. ^131^I-FIBG displayed a higher and more prolonged retention in tumors when compared to ^125^I-MIBG, which resulted in greater dose-dependent delay in tumor growth than with ^131^I-MIBG (Yamaguchi et al. 2018).

#### ^***18***^***F-FPOIBG***

An alternative strategy for the derivation of a fluorine-18 tracer from MIBG has been to introduce a fluoropropoxyl group onto the MIBG core structure, from which 4-[^18^F]fluoropropoxy-3-iodobenzylguanidine (^18^F-FPOIBG) was synthesized and compared with MIBG in three different NET-expressing cell lines. Uptake studies show that the tracer uptake at 2 h was, on average, lower than its lead MIBG, counting ^18^F-FPOIBG with10.2, 38.6, and 13.3%, and ^125^I-MIBG with 57.3%, 82.7%, and 66.3% in SK-N-SH, UVW-NAT and SK-N-BE(2c) cells, respectively. However, the corresponding bromine analogue of FPOIBG has demonstrated much higher uptake, with a higher IC_50_ value than either bromide or MIBG (Vaidyanathan et al. 2015). As a result, although FPOIBG does not provide superior in vivo properties than reported tracers, it offers a hint at the structure–activity relationships (SARs) of the tracers, which may prove useful for further tracer design, such as the intolerant iodine and the introduction of 3-fluoropropoxyl substitution consistent with *N*-[3-bromo-4-(3-fluoro-propoxy)-benzyl]-guanidine (LMI1195).

#### ^***18***^***F-LMI1195***

Prior to ^18^F-FPOIBG, ^18^F-LMI1195 was one of the first fluorine-18-labeled PET tracers derived from MIBG’s core structure without the direct introduction of fluorine-18 onto the benzene ring, avoiding the difficulty of radiofluorination. Different substitution patterns on the benzene ring/heterocycles, linkers between aromatic and guanidine moieties with or without β-hydroxyl group, as well as different substituted/integral guanidines have been investigated and patented by Lantheus Medical Imaging (Purohit et al. 2008). The chemical structure of LMI1195 was derived from MIBG with *meta*-bromine replacing iodine, with an extra *para*-3-fluoropropoxyl group for convenient radiolabeling and improved radiochemical yield compared to other MIBG derived tracers with direct radiofluorination on the electron-rich benzene ring. In this way, LMI1195 could be labeled from a brosylate precursor and a single-step fluorine-18 displacement reaction followed by HPLC separation (Yu et al. 2011). ^18^F-LMI1195 has demonstrated comparable in vitro properties to MIBG and more favorable in vivo characteristics than MIBG in both rats and rabbits, with clear and specific cardiac uptake and better heart-to-liver (H/L) ratios than MIBG (Yu et al. 2011). ^18^F-LMI1195 was used in imaging of regional cardiac sympathetic denervation in the rabbit model due to its high association with NET (Yu et al. 2012). Comparable to either MIBG or HED, LMI1195 has also showed high and specific accumulation in neuroendocrine tumors in the rat model, and more favorably than the reference MIBG (Gaertner et al. 2013). Following a successful phase 1 clinical trial, in which ^18^F-LMI1195 has demonstrated cardiac imaging potential with comparable radiation dose and favorable kinetics (Sinusas et al. 2014), it was compared with ^11^C-HED in a recent publication. The preliminary data suggested a highly correlated estimation of cardiac sympathetic innervation for both tracers, whereas ^18^F-LMI1195 showed significantly elevated heart-to-blood (H/B) ratios when compared to ^11^C-HED (Zelt et al. 2018). This leads into further phase 2 clinical trials for the prediction of arrhythmic events and the strategizing of the identification of patients who should receive ICD (NCT03493516).

While using rats and rabbits with and without sympathetic neuronal denervation, ^18^F-LMI1195 exhibited different uptake mechanisms due to species discrepancies: LMI1195 cardiac uptake did not change in the rat model but was greatly decreased in rabbits following treatment of uptake-1 selective blockade using DMI. This stems from the existence of extra-neuronal uptake (i.e. uptake-2) in the rat heart. A consistent result could be obtained from denervated rabbits and nonhuman primates (NHP), for the reason that in both animals only uptake 1 is responsible for the NET tracer uptake (Yu et al. 2013). Further assessment in rats using the non-selective uptake blocker phenoxybenzamine (PhB) confirmed the existence of both uptake 1 and 2 in rat hearts, where the cardiac uptake of ^18^F-LMI1195 could only be reduced by PhB, rather than DMI or saline control (Higuchi et al. 2013). First-pass tracer extraction fraction (EF) and washout were measured in an ex vivo study using isolated rabbit hearts, which is chosen due to comparable tracer uptake patterns in rabbits and humans. Results demonstrated a flow-dependent EF with DMI sensitivity. The irreversible vesicular monoamine transporter blocker reserpine pretreatment was unable to change the EF. Additionally, an electrical stimulation instead of DMI chase (DMI added in washout phase) was able to enhance the tracer washout rate, suggesting that the tracer storage and release mode at the nerve terminal was consistent with NE (Higuchi et al. 2015). The detailed kinetics of LMI11995 at the subcellular level have been confirmed in an in vitro study using both PC12 (vesicle-rich) and SK-N-SH (vesicle-poor) cell lines expressing NET. Both high-concentration KCl and reserpine were able to induce tracer washout due to storage turnover, as such an effect could be only observed in vesicle-rich PC12 cells but not in vesicle-poor SK-H-SH cells. Moreover, such KCl-induced spillover could be suppressed by the calcium chelator EDTA, which is consistent with the fact that the tracer release is mediated by a calcium influx resulting from membrane depolarization (Chen et al. 2018). Adding to these findings, a high-resolution autoradiography of rabbit heart slices following administration of both ^18^F-LMI1195 and ^201^Tl was performed, from which a homogeneous distribution of the former was observable throughout the LV wall. No significant differences were found when compared to the slightly discrepant transmural distribution of the latter tracer. Should ^18^F-LMI1195 be used in the clinic, the presented distinct characteristics in the LV distribution pattern should be considered as preliminary work for imaging result evaluation (Werner et al. 2019).

It is crucial that clinicians understand the characteristics of tracer uptake and retention kinetics when deciding on their suitable clinical applications. With this aim, ^18^F-LMI1195 was compared with ^123^I-MIBG and ^11^C-HED in rabbit hearts, where all three tracers exhibited DMI (pretreatment)-sensitive/neuronal-specific uptake. However, cardiac washout of ^11^C-HED, rather than ^18^F-LMI1195 and ^123^I-MIBG, was influenced by DMI chase (1.5 mg/kg administered 10 min after tracer injection), indicating a cycle of continuous NET uptake, and a ^11^C-HED release pattern at the nerve terminals, in contrast to the stable vesicle storage of the other two tracers (Werner et al. 2015). Considering the common benzylguanidine core structure of MIBG and LMI1195 (vs. the ephedrine of HED), it is possible to combine its chemical structure with its storage kinetics to conclude that the polar guanidine moiety retains the tracers in storage vesicles, whereas the relatively lipophilic monoamine structure of ephedrine can be released from these vesicles either passively or together with vesicle turnover.

#### ^***18***^***F-4F-MHPG and 3F-PHPG***

It is noteworthy that the insensitivity of known tracers to non-severe regional nerve loss delays possible early stage pharmaceutical intervention. Therefore, to overcome the rapid neuronal uptake and blood flow dependency of the mostly benzylguanidine-based radiotracers, and to provide physicians with a NET targeting tracer with more accurate regional denervation estimation, Raffel et al., synthesized over a dozen phenethylguanidine analogues as novel core structure of NET radiotracers, and investigated their neuronal uptake rate in isolated rat hearts. In this study, ^11^C-*N*-guanyl-*m*-octopamine (^11^C-GMO) showed a slow NET transport rate, with improved myocardial kinetics, when compared to HED. In cosntrast, ^11^C-*p*-hydroxyphenethylguanidine showed a rapid NET transport rate and rich accumulation in xenograft tumors in mice (Raffel et al. 2007). Further compartmental modeling and Patlak analysis of ^11^C-GMO kinetics in response to different doses of DMI in monkey hearts confirmed stable uptake, along with the possibility for providing robust and sensitive quantitative measurements of regional NET density (Raffel et al. 2013a). Meanwhile, several known tracers were evaluated in C6-hNET cells to determine their transport constants *K*_m_ and *V*_max_. Results have confirmed a strong linear correlation between NET-mediated neuronal transport rates and their in vitro transport parameters, as given by the ratio *V*_max_*/K*_m_*.* This project provided useful insights into tracer structure design and NET transport constants (Raffel et al. 2013b). Based on this knowledge, ^18^F-4-fluoro-*m*-hydroxyphenethylguanidine (^18^F-4F-MHPG) was discovered. It features comparable biodistribution to MIBG and HED in rats, along with slow cardiac uptake and long-term retention kinetics in isolated rat hearts. Further NHP PET study has proved its feasibility for cardiac imaging by allowing for more accurate quantification of regional cardiac sympathetic nerve density (Jang et al. 2013a). A detailed biodistribution study revealed low uptake in the lungs and liver, in addition to high quality and DMI-sensitive cardiac imaging. Uptake in adrenal glands has also proved its potentiality for oncological application (Jang et al. 2013b). With the establishment of the new synthetic approach to ^18^F-hydroxyphenethylguanidines, ^18^F-3-fluoro-*p*-hydroxyphenethylguanidine (^18^F-3F-PHPG), the structural isomer of ^18^F-4F-MHPG, has been evaluated (Fig. 1a). In monkey studies, comparable properties between^18^F-3F-PHPG and ^18^F-4F-MHPG have been demonstrated, showing higher H/B (4.0 vs. 3.0) but slightly lower H/ L ratio (2.2 vs. 2.5) due to the relatively faster liver clearance of the latter tracer. Fast clearance of these tracers from the blood has been revealed in monkey studies with sulfate as the main metabolite of ^18^F-4F-MHPG, but results are yet unclear for ^18^F-3F-PHPG. The mean time to 50% intact radiotracer is 6.7 and 2.3 min (Jung et al. 2017). As a result, both ^18^F-4F-MHPG and ^18^F-3F-PHPG have been evaluated in healthy human subjects, from which the information of not only the biodistribution and metabolism, but also, more importantly, the potential of quantitative estimates of regional cardiac sympathetic nerve density could be obtained. These results are consistent with the data obtained from the monkey study, with both excellent imaging properties and individual kinetic characteristics, such as different neuronal accumulation time and liver clearance rate (Raffel et al. 2018). A further study involved eight patients with HF who underwent cardiac innervation PET scans with both tracers while using ^13^N-ammonia as a reference. The results showed that both tracers are able to provide reliable quantitative metrics of regional sympathetic nerve density and could be used to strategize HF patients for ICD placement (Raffel et al. 2017).

**Fig. 2:**
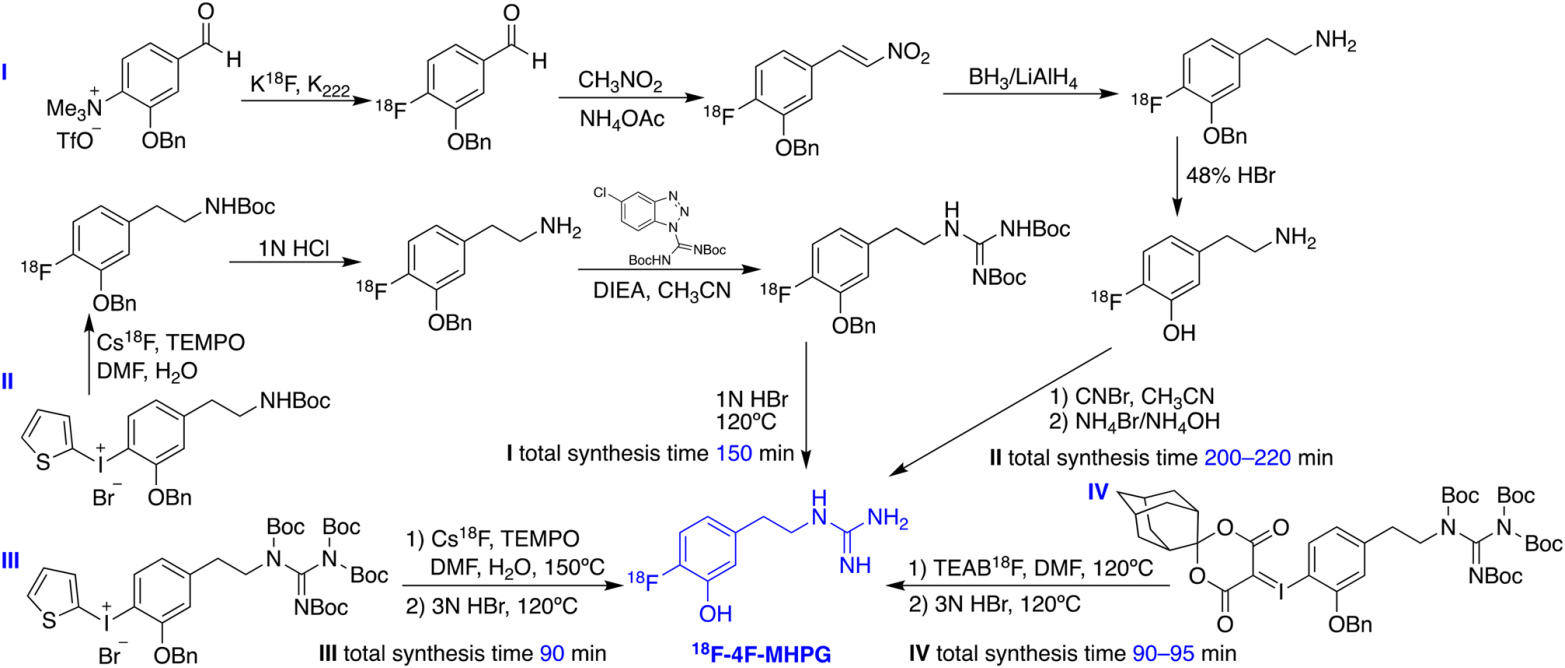
Development of the synthetic scheme for the radiolabeling of ^18^F-4F-MHPG (Jang et al. 2013a, b; Jung et al. 2017, 2019). This figure was created using ChemDraw 16 for Mac

Similar to the case of ^18^F-MFBG/PFBG, the radiolabeling of phenethylguanidine tracers started from using radioisotope carbon-11 for preliminary evaluation, to build up the correlation of chemical structures with NET affinity. Due to the aforementioned disadvantages of carbon-11 tracers, however, later stage development mainly focused on fluorine-18-labeled ones, as exemplified by ^18^F-4F-MHPG (Fig. 2). The initial radiofluorination procedure required four steps because of the requirement of the electron-withdrawing group on the benzene ring (Jang et al. 2013a), but was soon simplified to four steps but one pot reaction including the fluorination from 2-thienyl iodonium salt and the following formation of the guanidine moiety (Jang et al. 2013b). Although the method has been improved, multistep radiolabeling is still time consuming with low radiochemical yield that is not suitable for clinical application. As a result, this research group has kept the iodonium salt precursor but used *tetra*-Boc as protecting groups on the guanidinyl moiety to accomplish two-step-one-pot radiolabeling. The procedure was applied to both ^18^F-4F-MHPG and 3F-PHPG, with an average yield of final product up to 7.0% and 8.0%, respectively (Jung et al. 2017). In exploring new methods that offer higher yield, spirocyclic iodonium ylide rather than 2-thienyl iodonium salt precursor has been used due to the even higher electron density offered by the former moiety, which could lead to higher yield. Under optimized automated reaction conditions, the yield increased threefold, averaging 7.8%, which the authors claimed being well suited to on-site production for clinical PET studies (Jung et al. 2019).

#### ^***18***^***F-AF78***

**Fig. 3:**
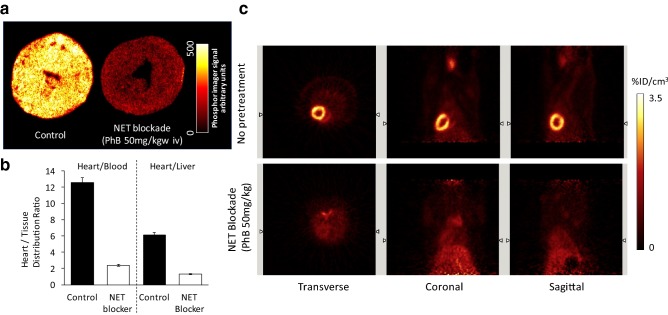
**a** Autoradiography of left ventricular short axis slices from rats following administration of ^18^F-AF78 with (right) and without (left) NET blockade. **b** Tissue distribution ratios of ^18^F-AF78 with (hollow column) and without (solid) NET blockade. **c** Static PET images of the uptake of ^18^F-AF78 in rats with (bottom) and without (top) NET blockade 10 min before radiotracer administration (Chen et al. 2019). This figure was created using Microsoft PowerPoint version 15.38 for Mac then pasted as PDF format into Word file

Although ^18^F-4F-MFBG/3F-PFBG and^18^F-FIBG are superior MIBG analogues, their potential clinical applications are limited due to the nature of their chemical structures—the direct radiofluorination on the electron-rich benzene ring is normally not suitable for single-step fluorine-18-labelling. Consequently, a long and demanding radiolabeling procedure along with low radiochemical yield has made further development challenging. To investigate the relationships between the chemical structures of radiotracers and their affinity for NET, our research group has recently developed a new NET-targeting PET tracer: ^18^F-1-(3-fluoro-4-(3-fluoropropoxy)phenethyl)guanidine (^18^F-AF78). It bears a phenethylguanidine core structure of ^18^F-3F-PHPG for potential slow uptake kinetics, but with an easier radiolabeling procedure and a high yield (27%) as a result of the introduction of a 3-fluoropropoxy substitution—a strategy to develop ^18^F-LMI1195 from ^123^I-MIBG as mentioned above (Chen et al. 2019). Cell uptake studies of ^18^F-AF78 have demonstrated an almost identical affinity for NET as NE and MIBG. Both ex vivo autoradiography of the rat heart and in vivo imaging in rats showed homogeneous and specific cardiac uptake throughout the left ventricular wall (Fig. 3). High H/B and H/L ratios (12.54 ± 0.53 and 6.14 ± 0.35) have also been observed (Chen et al. 2019), and are comparable to those of ^123^I-MIBG (11.91 ± 1.60 and 2.42 ± 0.41) and ^18^F-LMI1195 (15.56 ± 3.61 and 6.21 ± 1.68) (Yu et al. 2011). Time–activity curves of ^18^F-AF78 in rats have demonstrated stable and long-term cardiac uptake with favorable H/L ratios following 10 min of tracer injection, due to the fast liver uptake washout (Fig. 3). Currently, the evaluation of AF78 in different animal species is still ongoing for further in vivo characteristics.

Of note, while developing AF78 from 3F-PHPG, its isomer AF51, the corresponding 3-fluoropropoxy derivative of 4F-MHPG, was also synthesized and evaluated in cell uptake assay as well. It demonstrated a great loss of NET affinity, which provides an insight into the SARs of these NE-derived tracers. To combine the drop of activity of derivation from MIBG to FPOIBG as mentioned above, a substituent larger than iodine on the *meta*-position would not be tolerable. Consequently, a loss of activity due to the introduction of a 3-fluoropropyl group to 4F-MHPG would be reasonably explained. The reason for choosing 3-fluoropropyl over 2-fluoroethyl is its in vivo stability, as the latter metabolizes faster through defluorination (Kuchar and Mamat 2015). Possible byproduct, such as the formation of vinyl from precursors during radiofluorination, could also be prevented. Furthermore, the ether that substitutes the phenol might change the kinetics and metabolism of the radiotracer in vivo, as it has been proved that one of the major metabolites of 4F-MHPG is the sulfate of the phenol (Jang et al. 2013b). In addition, ether is more stable against oxidation than phenol, which might be the case of the presumable metabolite of 3F-PHPG (Jung et al. 2017). Lastly, cardiac uptake may be also related to the chemical properties of these radiotracers. For example, LogP, or polar surface area, which can be determined by comparing the biodistribution data of 4F-MHPG with MIBG and HED (Jang et al. 2013a). Of note, when evaluating the biodistribution and kinetics of ^18^F-AF78 in different animal species, cardiac uptake showed significant differences (unpublished data): it showed different modes of cardiac uptake in animals with only the uptake-1 mechanism, i.e. the uptake can be fully blocked by DMI; and those present both uptake-1 and uptake-2/extra-neuronal uptake, where the radiotracer uptake can only be fully blocked by PhB. These results are in accordance with the data obtained from assessing ^123^I-MIBG, ^18^F-LMI1195 and ^18^F-MHPG/PHPG in different animal models (Rischpler et al. 2013; Higuchi et al. 2013; Jang et al. 2013b; Jung et al. 2017): these radiotracers would be preferably taken up in the heart via uptake-2/extra-neuronal uptake mechanism in animals, such as rats and pigs, where both uptake-1 and -2 mechanisms present. In contrast, in uptake-1 dominant animals, such as rabbits and monkeys, as well as in humans, these tracers are primarily taken up in the heart via NET-mediated uptake-1 mechanisms.

### Radiotracers targeting NET in central nervous system

As mentioned in the introduction section, malfunction of the noradrenergic system in CNS also plays an important role in the process of neurodegenerative diseases, depression, ADHD, and obesity (Borodovitsyna et al. 2017). Therefore, it is conceivable that NET-targeting radiotracers could be used to monitor the function of the noradrenergic system in the CNS, provide diagnostic information concerning the process of these diseases, and instruct therapeutic applications. However, although all the abovementioned NET-targeting radiotracers can be used for peripheral SNS imaging, they are unable to penetrate the blood–brain barrier (BBB), most probably due to their polar structures with guanidine moieties. As a result, several NET-targeting and BBB-penetrating radiotracers have been developed based on clinically used and NET-selective antidepressants.

#### ^***11***^***C-ME@HAPTHI***

Rami-Mark et al. (2013) reported a scaffold using a benzothiadiazole core structure (Fig. 1b) to obtain (S)-1-(3-hydroxy-4-([^11^C] methylamino)butyl)-3-phenyl-1,3-dihydrobenzo[c][1,2,5]thiadiazole 2,2-dioxide (^11^C-Me@HAPTHI) based on their previously reported ^11^C-Me@APPI. Following successful preparation and radiolabeling, it has displayed good affinity and selectivity for NET over the dopamine transporter (DAT) and serotonin transporter (SERT or 5-HTT), which is crucial for the accuracy of CNS imaging. It is expected to pass the BBB based on the in vitro data. Furthermore, the tracer shows excellent metabolic stability in human liver microsomes with > 99% intact after 60 min incubation. Autoradiography on human brain tissue has revealed NET-specific uptake in NET-rich regions (Rami-Mark et al. 2015). Notably, in addition to CNS uptake, significant cardiac uptake of ^11^C-Me@HAPTHI has been observed, albeit with a more lipophilic chemical structure than abovementioned radiotracers. After transient ischemia, reduced ^11^C-Me@HAPTHI uptake was detected in rat hearts from day 1 to 4 weeks, confirmed by an increased uptake ratio of ischemic area to the non-ischemic area, and increased uptake ratio of non-ischemic area to the blood pool. A reduced ratio of cardiac uptake to blood radioactivity by the pre-injection of blockers confirms its high specificity. Slightly reduced apical ^18^F-FDG uptake was observed in the left ventricle, revealing reduced myocardial viability with corresponding sympathetic nerve denervation in the heart, as indicated by ^11^C-Me@HAPTHI uptake in the ischemic region (Li et al. 2016). These preliminary data have proved the feasibility of cardiac imaging using different core structures, opening a door to the development of a new series of radiotracers for the diagnosis of cardiac innervation, and reducing the effect of the subcellular conditions of the sympathetic nerve terminals. The fact of being a carbon-11-labeled tracer might prevent its further application, but several ways have been proven possible for the modification of this tracer into a fluorine-18-labeled one with improved properties.

#### ^***11***^***C-MRB***

(S,S)-[^11^C]*O*-methyl-reboxetine (^11^C-MRB) is the methyl analogue of reboxetine (Fig. 1b), a specific NET inhibitor and antidepressant against panic disorders and ADHD. The enantiomeric pure tracer was yielded after resolution via chiral HPLC (Lin and Ding 2004). Only (S,S)-^11^C-MRB (IC_50_ 2.5 nM vs. 85 nM of (R,R)-isomer) shows strong DMI sensitivity and high specific uptake in the NET-rich regions of the brain and heart in baboons (Ding et al. 2003). It shows higher specific binding to NET than other NET inhibitor-derived ligands, such as nisoxetine or oxaprotiline (Ding et al. 2005). In addition, ^11^C-MRB has a sixfold higher affinity for NET than SERT. Although there are conflicting results based on a baboon study concerning the sensitivity and reliability of its utility as a PET radiotracer (Severance et al. 2007), ^11^C-MRB studies in rhesus monkeys have demonstrated its suitability for the determination of NET occupancy, using dose-dependent inhibition by infusion of the NET inhibitor atomoxetine (Gallezot et al. 2011). The first human study performed involved 24 male subjects. The NET density determined by ^11^C-MRB PET in these subjects follows the order of locus coeruleus, thalamus, and caudate nucleus, which is in accordance with the results obtained from NHP (Logan et al. 2007).

Following the confirmation of its feasibility, ^11^C-MRB has been applied to monitor NET density in the CNS under pathological conditions in several different fields, including obesity, ADHD, and PD. Noradrenergic dysfunction and impaired NE clearance are implicated in obesity. Consequently, the researchers using ^11^C-MRB PET have come to mutual conclusions that the NET availability in the thalamus region of obese adults is decreased when compared to healthy controls (Li et al. 2014; Bresch et al. 2017). In addition, by measuring the CNS NET availability, it is possible to predict the result of a dietary intervention, and is helpful for adjusting treatment options of highly obese adults (Vettermann et al. 2018). BAT plays a role in energy balance and is also regulated by the SNS. After an initial evaluation of ^11^C-MRB in rats, tracer uptake in BAT, with proved specificity under basal, room temperature conditions, was observed to be threefold higher than that of ^18^F-FDG. However, the latter one requires cold stimulation for good imaging quality (Lin et al. 2012). The same result could be replicated in humans, showing ^11^C-MRB uptake in BAT to be equally evident under both room temperature and cold conditions, though with gender differences (Hwang et al. 2015). When ^11^C-MRB PET was further applied to monitor BAT only in healthy women, findings were consistent with reports that, as mentioned above, NET is decreased in obesity, which suggests that sympathetic innervation of BAT is altered in obesity (Sanchez-Rangel et al. 2019). ADHD is a heterogeneous disorder with NET as the key target for treatment. A PET-MRI study using ^11^C-MRB has successfully demonstrated that, rather than morphological changes, there is a decrease in NET availability in fronto–parietal–thalamic–cerebellar regions in the brains of ADHD patients (Ulke et al. 2019). Cardiac ^123^I-MIBG scintigraphy has been proposed for early detection of PD, but its feasibility as a routine clinical practice is limited (Skowronek et al. 2019). The potential of evaluating PD patients using ^11^C-MRB PET has been confirmed, and the imaging protocol has been optimized following the investigation of kinetic models. An acquisition time of 30 min after 50 or 60 min of tracer injection allows for a reliable estimation of NET, which can significantly reduce the discomfort of PD patients (Brumberg et al 2019). Lastly, in addition to the above applications, ^11^C-MRB has also been used to investigate noradrenergic activation and modulation in different regions of the brain in response to insulin-induced hypoglycemia (Belfort-DeAguiar et al. 2018).

#### ^***18***^***F-FMeNER-D***_***2***_

^18^F-FMeNER-D_2_ is a fluorine-18-labeled CNS NET tracer that is the di-deuterated analogue of ^18^F-FMeNER, developed to improve its in vivo stability by reducing the defluorination (Fig. 1b). A preliminary study in rhesus monkeys demonstrated fast and long retention of ^18^F-FMeNER-D_2_ in the thalamus and brainstem (Takano et al. 2009). It is prepared by *O*-fluoromethylation of desfluoromethoxy-(S,S)-FMeNER with ^18^F-di-deutero-bromofluoromethane. After optimization, fully automated radiolabeling can be performed, yielding 1.0–2.5 GBq of formulated tracer within 95 min (Rami-mark et al. 2013). It initially shows DMI-sensitive binding to NET in monkeys and in ex vivo autoradiography on post-mortem human brains. The tracers are localized in the locus coeruleus and thalamus (Schou et al. 2004; 2005). An initial in vitro investigation of the tracer revealed stability against MAO and catechol-*O*-methyl transferase (COMT), along with fast metabolism by CYP450 enzymes (Rami-Mark et al. 2016). An evaluation on the post-mortem AD brain tissue with autoradiography showed a significant decrease in NET densities in various brain regions (e.g. thalamus), indicating the possible use of ^18^F-FMeNER-D_2_ PET as imaging biomarker in AD (Gulyás et al. 2010).

Takano et al. (2008a, b) evaluated the tracer in healthy subjects, which showed a good radiation tolerance in the entire body, and especially good brain penetration and selective retention in NET-rich regions. A further study indicated that combining the radiotracer with a template method can map the distribution of NET in different regions of the brain. Functional regions of interest (ROIs) with higher nondisplaceable binding potential (BP_ND_) thresholds yielded higher BP_ND_ and lower coefficients of variance than did anatomical ROIs (Takano et al. 2008c). The regional distribution of the tracer is highest in the thalamus and lowest in the caudate, which is in accordance with the results demonstrated by previous PET and post-mortem studies of NET on human brain tissue. This opens up the potential of noninvasive estimation of NET occupancy by antidepressants (Arakawa et al. 2008).

In recent years, with the solid potential of NET imaging in the CNS being proven, ^18^F-FMeNER-D_2_ has been used in several clinical studies focused on the quantification of NET density in ROIs, along with its changes in different related diseases. However, quantification of NET in the cerebral cortex has been difficult due to the unfavorable kinetics of ^11^C-MRB and the defluorination of ^18^F-FMeNER-D_2_, along with the disadvantages due to the short half-life of carbon-11-labeld tracers. After systemic evaluation of the tracer distribution in ten healthy volunteers, Moriguchi et al. established methods for quantifying NET densities in the brain through the performance of 90-min dynamic scans, which then provided a sufficient amount of data while being unaffected by the defluorination that normally begins to increase beyond 120 min (Moriguchi et al. 2017a).

The NET has been suggested to play a crucial role in major depressive disorder (MDD), but its availability in this disease and its relationship with clinical symptoms are not clear. A 19-patient MDD study using ^18^F-FMeNER-D_2_ PET revealed a positive correlation between BP_ND_ values in the thalamus region and altered attention in the patients (Moriguchi et al. 2017b). As it binds to NET (i.e. NET targeting), ^18^F-FMeNER-D_2_ can, therefore, not only reveal the distribution and density of CNS NET but also show NET occupancy by antidepressants or antipsychotics. Presently, investigated ligands include nortriptyline (Sekine et al. 2010), clomipramine (Takano et al. 2011), quetiapine (Nyberg et al. 2013), milnacipran (Takano et al. 2013; Nogami et al. 2013), nortriptyline (Takano et al. 2014), and duloxetine (Moriguchi et al. 2017c), which compete for the transportation via NET. In all cases, the estimated administration dose, as well as plasma drug concentration, can be obtained to guide the drug application. The NET occupancy measurement may provide information regarding the spectrum of efficacy of an antidepressant. Some reported antidepressant drugs involve blockade of both SERT and NET. Using ^18^F-FMeNER-D_2_ PET measurements, it has been proved that venlafaxine occupies 8–61% of NET in a dose-dependent manner, as seen in a study involving 12 major depressive disorder patients who had responded to venlafaxine (Arakawa et al. 2019a). Thus, ^18^F-FMeNER-D_2_ PET can also be used to predict the potential of certain drugs as an antidepressants, exemplified by the research on tramadol in NHP (Arakawa et al. 2019b).

In contrast to the application of ^11^C-MRB in ADHD, a study on ^18^F-FMeNER-D_2_ PET by Vanicek et al. (2014) found no significant difference in either NET availability or distribution in ADHD-relevant regions of the brain. However, Sigurdardottir et al. (2016) used it to identify the genotype-dependent difference in NET BP_ND_ between healthy controls and ADHD patients. It provided further evidence of NET imbalance in several brain areas, pointing to epigenetic dysfunction in ADHD (Sigurdardottir et al. 2019).

#### ^***18***^***F-NS12137***

Another CNS fluorine-18-labeled tracer is exo-3-[(6-[^18^F]fluoro-2-pyridyl)oxy]-8-azabicyclo[3.2.1]-octane (^18^F-NS12137), which is derived from ^11^C-NS8880 (Vase et al. 2014), and has shown good selectivity over SERT and DAT (Fig. 1b). It has been successfully radiolabeled using both the ^18^F-F_2_ and ^18^F-selectfluor methods, with radiochemical yield 3.9% and 10.2%, respectively (Kirjavainen et al. 2018). The radiolabeling method was then optimized using a copper-mediated pathway and stannane precursor to obtain ^18^F-NS12137 with 15.1% yield and up to 300 GBq/µmol specific activity (Lahdenpohja et al. 2019a, b). Further optimization of the fluorination using a bromide precursor led to 18.6% yield and > 500 GBq/µmol specific activity (López-Picón et al. 2019). Its uptake in NET-rich areas of the rat brain was demonstrated with specificity proven by pretreatment of nisoxetine, a known NET antagonist. Defluorination and slow increase of bone uptake could be observed after time (Kirjavainen et al. 2018). PET imaging in adult and immature Sprague–Dawley rats, along with ex vivo studies using autoradiography, has demonstrated uptake of the radiotracer in brain areas rich in NET, especially the locus coeruleus, a quite small volume for PET imaging. The tracer shows optimal characteristics (such as fast washout and high specificity) that are favorable for NET imaging in the brain (López-Picón et al. 2019).

## Discussion

### NET targeting and NET-function targeting tracers

The term “NET targeting” has always been used when discussing the diagnosis of SNS in the heart, both in other reviews and in the present one. However, it is worth mentioning that all the cardiac radiotracers described above are actually “NET function targeting”, because they will be taken up by NET and transported into the neurons, particularly granular vesicles, with the exception of ^11^C-HED as discussed above (Werner et al. 2015). Therefore, the PET signal reflects the transportation/density of NET, which relies on the structural integrity of the sympathetic neurons. In addition, there are also NET structure-targeting radiotracers, mostly derived from antidepressants and mainly used in the imaging of NET density in the CNS. The rationale behind this is the lipophilicity of these compounds. On the one hand, this allows them to easily pass the BBB, on the other hand, it may lead to high liver uptake that is not ideal for cardiac imaging due to the scattering effect. As a result, there is almost a clear-cut boundary between cardiac NET tracers and CNS NET tracers: the former ones are majority NET function-targeting tracers with polar benzylguanidine or phenethylguanidine structures, whereas the latter ones are mostly derived from known NET-selective antidepressants with BBB penetration properties. Researchers have attempted to modify the chemical structure of MIBG by introducing 1,4-dihydroquinoline as chemical delivery system to improve its uptake in the brain, but the result requires further optimization (Gourand et al. 2019). The structural–functional difference is due to the different properties between the heart and the brain. Assessment of sympathetic innervation depends on both the blood flow—through which the tracer reaches the target tissue—and the NET uptake kinetics. Subsequently, most known radiotracers, such as MIBG and LMI1195, are quickly transported into the neurons and therefore cannot be used to quantify NET density precisely. The design rationale of 3F-MHPG and 4F-PHPG by researchers from University of Michigan counteracted this issue by introducing tracers with slow-uptake mechanisms, which has been proven in ex vivo rat heart studies, and in NHP (Raffel et al. 2007). Clinical trials of these tracers are still ongoing, and further proofs of the concept are needed.

### NET tracer specificity and selectivity

In this review, we have provided an overview of the currently reported NET radiotracers derived from its natural substrate, NE, or NET-selective antidepressants. This has included not only their preclinical evaluation and clinical application, but also their potential use in the fields other than heart and neuroendocrine tumors. Several carbon-11 and fluorine-18-labeled tracers derived from antidepressant drugs with chemical structures different from cardiac ones have been used in the investigation of CNS imaging, in the context of ADHD and PD, for example. The advantages of using carbon-11 radioisotope are: the easy access to their corresponding precursors, along with their unchanged chemical structures and biological properties in vivo, particularly when the CNS is involved, as the development of new structures by chemists requires much longer time. In addition, the new generation of fluorine-18-labeled PET tracers, such as ^18^F-LMI1195 and ^18^F-4F-MHPG/3F-PHPG, has already reached clinical trials, and demonstrated excellent imaging quality and spectacular kinetic advantages of quantifying regional innervation. Furthermore, the distinctive slow neuronal uptake and long-term neuronal retention in the heart represented by the phenethylguanidine series of NET-function targeting tracers has provided clinicians with more possibilities for quantitatively measuring regional denervation conditions. Thus, from the classical SPECT tracer ^123^I-MIBG, with its benzylguanidine core structure, to the latest easily labeled PET tracer, ^18^F-AF78, more details regarding the structure–activity relationships of these NET-function targeting radiotracers have been revealed, which is helpful for the design of new generation PET tracers.

**Fig. 4:**
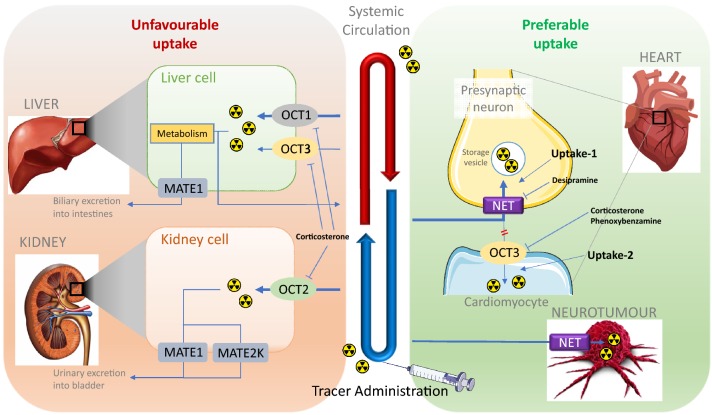
Illustration of simplified NET-targeting radiotracer biodistribution. This figure was created using Microsoft PowerPoint version 15.38 for Mac then pasted as PDF format into Word file

However, it should be mentioned that the currently available radiolabeled tracers or therapeutic agents are not selective to NET transport function. In the human or animal body, several extra-neuronal uptake mechanisms, or non-adrenergic uptake, such as DAT and SERT uptake, also exist and are responsible for the extraction and excretion of these radiotracers into or through different organs, which may lead to unfavorable uptake and corresponding side effects. Due to the fact that either the guanidine moiety of the MIBG-derived NET-function targeting tracers or antidepressant-derived NET-targeting CNS tracers would be in cation form under physiological conditions, organic cation transporters (OCTs) are considered to contribute most to these extra-neuronal uptake mechanisms (Fig. 4). Among the subtypes of the OCT family, OCT1 and OCT2 are critical for the elimination of a wide array of drugs and environmental toxins and are restricted mainly to excretory organs, dominantly in the liver and kidney, respectively (Solbach et al. 2011), which may be the molecular basis of the liver or kidney uptake of radiotracers. OCT3 transports a wide range of monoamine neurotransmitters, hormones, and steroids and shows a much broader tissue distribution, including skeletal muscle, heart (Solbach et al. 2011), brain (Wu et al. 1998; Gasser and Lowry 2018), and placenta (Chen et al. 2010). Although OCT3 has a major expression in the brain, it is unlikely that the currently reviewed tracers with guanidine moieties will be able to penetrate the BBB due to their very polar guanidine structures. Such radiotracers targeting NET, yet mediated by OCT3 uptake in the CNS, would be prudently considered.

Until now, although several radiotracers targeting NET transport-function have been reported, no NET-selective compound has been reported and no such agent has been approved for clinical application. All the research focus is on either NET or OCTs alone, but without correlating the OCT-dependent side effects to improve the NET-selectivity for higher accuracy diagnosis and higher efficiency cancer therapy. As the only NET-function targeting tracer currently applied clinically, ^123/131^I-MIBG is not exclusively selective towards NET but can be also taken up by OCTs in both animal models and humans. Consequently, its uptake might not accurately reflect the SNS function of the heart or properly affect cancer therapy, which urges the development of radiotracers with higher NET selectivity over OCTs, for better imaging accuracy and therapeutic specificity. Ito et al. (2012) have already proven MIBG uptake via hOCT1 and hOCT2 in HEK295 cells. First, less NET-selective radiotracers with intensive liver uptake, presumably via OCT1, decrease the H/L contrast and make it difficult to interpret the condition of the heart in the LV inferior wall. Photon scattering from high liver radioactivity accumulation remains a challenge for the diagnosis of heart diseases (Kim et al. 2010). Second, researches, such as ^123^I-MIBG imaging involving therapy-resistant hypertension patients before and after catheter-based renal sympathetic denervation or kidney autotransplantation (Dobrowolski et al. 2015, 2018), have proven the uptake of MIBG into kidney irrelevant to sympathetic nerve activity in the kidney, presumably due to its non-specific uptake via OCT2 or other transporters. Thus, a NET-selective tracer would open a new door for the diagnosis of renal sympathetic innervation. Furthermore, decreased OCT activity of NET-targeting radiotracers may reduce the fast excretion through kidneys and the rapid urinary elimination, which has always resulted in the bladder being the actual critical organ for imaging or therapy. Therefore, a higher NET selectivity might reduce the speed of excretion, which would consequently reduce the overall dose administration and the corresponding unnecessary off-target radiation exposure. Third, in addition to the specific tumor uptake required for diagnostic and therapeutic applications, non-tumorous tissues also incorporate MIBG through OCTs (Bayer et al. 2009, 2016), which will reduce the effective radiation dosage for the target organ while causing an unnecessarily high dose utilization of radioactivity and delayed toxicity in these related organs. It has been reported that with pretreatment of hydrocortisone, the liver and heart uptake of MIBG in patients was reduced due to the blocking of OCTs (Bayer et al. 2016). Therefore, it is conceivable that with NET-selective radiotracers and derivatives, such additional drug intervention can be prevented and the radiation dose required could be further decreased, especially when juvenile or elderly patients are involved (Agrawal et al. 2018). It is crucial to summarize the SARs of the radiotracers selectively targeting NET over OCTs using different cellular- and animal-based models, which is helpful for elucidating the role of OCTs in drug design and kinetics optimization.

A NET tracer might also be taken up by other monoamine transporters, such as DAT and SERT. Both play very minor roles in the heart as far as it is reported. However, in the CNS, it is crucial to investigate the selectivity of radiotracers targeting CNS NET, both in cell-based assays and in animal studies. Therefore, a blocking study, i.e. a pretreatment with DMI that significantly reduces the uptake of potential NET tracers, is always performed in all in vitro and in vivo studies. DMI has been proven as a NET selective blocker with minor effects on SERT (Biaggioni and Robertson 2012; Owens et al. 1997; Tatsumi and Groshan 1997). Further study in a rabbit model confirmed that the dosage required to inhibit MIBG uptake is much higher with escitalopram than with DMI. These results proved that MIBG uptake is selective to NET over SERT (Werner et al. 2018). In an early study investigating MIBG uptake by sympathetic neurons, the human NET, bovine DAT, and the rat SERT were cloned and expressed in various cell lines. The results also demonstrated nanomolar-range inhibition of MIBG uptake by DMI, with no significant uptake by bovine DAT or rat SERT (Glowniak et al. 1993). As a result, it is reasonable to believe that these reported NET tracers are relatively selective to NET over DAT and SERT (Table 1). As discussed in the last section, most of the cardiac NET tracers are NET function targeting ones, which are stored in granular vesicles. Therefore, most of them show a VMAT2 affinity that allows them to be transported into the vesicles. It is difficult to summarize the pharmacological data of all reported NET tracers, since the in vitro and in vivo studies are normally performed in a large variety of cell lines, tissues, and animal species. Many targets were not considered while developing these tracers. Therefore, the results cannot be clearly presented using accurate numbers, such as an IC_50_ values. For the reader’s convenience, we attempted to organize the related data of selected NET tracers into a table containing the transporters discussed in the current review (Table 1). Among them, MIBG has received most of the attentions and has been investigated at a variety of targets relatively thoroughly over 30 years of clinical application.

**Table 1:** The activity of selected NET radiotracers at various transporters

Radiotracer	NET	DAT	SERT/5HTT	VMAT2	OCTs	PMAT
^123^I-MIBG	(+) in human neuroblastoma cells (Montaldo et al. 1991; Glowniak et al. 1993; Graefe et al. 1999) (+) bovine chromaffin granule membrane (Gasnier et al. 1986)	(−) in monkey kidney cells (Glowniak et al. 1993)	(+) in humans (Hanson et al. 1989) (−) in monkey kidney cells (Glowniak et al. 1993) (+) in human platelets (Rutgers 1993) (+) in rabbit platelets (Saihkay et al. 2011) (−) in neuroblastoma and rabbit (Werner et al. 2018)	(+) in GOT1 and BON cells (Kölby et al. 2003) (+) bovine chromaffin granule membrane (Gasnier et al. 1986)	( +) human and mouse OCTs in HEK293 (Ito et al. 2012) ( +) in neuroblastoma (Bayer et al. 2009,2016)	ND
^11^C-HED	(+) in C6 rat glial cells (Foley et al. 2002)	(–) in tenfold less than NET (Foley et al. 2002)	(−) in human platelets (Foley et al. 2002)	(−) in bovine chromaffin vesicles, 56-fold less than NET (Foley et al. 2002)	ND	ND
^18^F-LMI1195	(+) in human neuroblastoma cells (Chen et al. 2018)	ND	ND	(+) indirectly through reserpine releasing (Chen et al. 2018)	( +) indirect proof of uptake-2 in rat heart (Higuchi et al. 2013)	ND
^18^F-4F-MHPG ^18^F-3F-PHPG	(+) in isolated rat heart, in monkey (Jang et al. 2013; Jung et al. 2017)	ND	ND	(+) sensitive to reserpine in isolated rat heart (Raffel et al. 2007; Jung et al. 2017)	ND	ND
^18^F-AF78	(+) in human neuroblastoma cells (Chen et al. 2019)	ND	ND	ND	ND	ND
^11^C-MRB	(+) in vivo (Melloni et al. 1984) (+) in mice brain (Ghose et al. 2005)	(?) in baboon brain (Logan et al. 2005) (−) (Melloni et al. 1984) (−) in mice brain (Ghose et al. 2005)	(?) in baboon brain (Logan et al. 2005) (−) in vivo (Melloni et al. 1984) (−) in mice brain (Ghose et al. 2005)	ND	ND	(?) reboxetine ( +) at hPMAT (Haenisch and Bönisch 2010)
^18^F-FMeNER-D_2_	(+) in monkey (Schou et al. 2004,2005) (+) in MDD patients (Nogami et al. 2013)	ND	ND	ND	ND	ND
^18^F-NS12137	(+) in rat brain (Kirjavainen et al. 2018)	ND	ND	ND	ND	ND

(+) represents positive affinity of the corresponding tracer at the target; (−) represents none or much lower affinity than NET; (?) represents unclear conclusion; ND represents not determined

### Limitations of the current strategy for the development of cardiac NET tracers

Currently, there are three categories of cardiac NET tracers: (1) those derived directly from NE; (2) those derived from MIBG with a benzylguanidine core structure; (3) those with a phenethylguanidine core structure. The tracers from category 1, exemplified by ^11^C-ephedrine and ^11^C-phenylephrine, are not metabolically stable against MAO. On the one hand, the guanidine moiety in categories 2 and 3 improves not only the stability of these tracers against MAO, but also increases the hydrophilicity that is helpful for reducing liver uptake where nonpolar structures are preferred. On the other hand, the guanidine structure, as suggested by the investigation of metformin, is the substrate for OCT1 and OC2. Thus, unfavorable uptake in the off-target tissues increases, as discussed in the last section. Furthermore, NET structure-targeting tracers derived from antidepressants are not preferable in the cardiac imaging due to their high lipophilicity and consequent high liver uptake. ^11^C-Me@HAPTHI is one of the few nonpolar tracers that shows both CNS and cardiac NET uptake ability. However, such research is still in its preliminary stages and, to form a more objective judgment, it is necessary to address the changing of the radionuclide from carbon-11 to fluorine-18, as well as properties such as organ selectivity, in vivo stability and kinetics. In addition to the abovementioned tracers, ^11^C-HED, with its metaraminol structure, is a special type of NET tracers. It has an amine instead of guanidine-based structure yet is still MAO stable due to its *N*- and *α*-methyl substituents. It is suggested to be taken up via uptake-1/NET selectively, possibly with low affinity at OCT3. Nevertheless, in addition to the disadvantages stemming from carbon-11-labeling, its special storage mode—constant leaking and reuptake—makes the calculation of parameters reflecting integrity of NE turnover difficult. In summary, there are still areas for improvement in designing an optimal NET tracer with NET specificity and ideal in vivo kinetics. A thorough investigation on the ligands and radiotracers targeting NET will provide useful information as well as a paradigm for obtaining highly selective NET-targeting theranostic ligands with selectivity over other transporters, especially OCTs.

## Electronic supplementary material

Below is the link to the electronic supplementary material.

Supplementary file1 (CDX 94 kb)

## Abbreviations

1TC: One-tissue compartment
5-HTT: 5-Hydroxytryptamine transporter, also SERT
AD: Alzheimer’s disease
ADHD: Attention-deficit hyperactivity disorder
ADMIRE-HF: ADreview Myocardial Imaging for Risk Evaluation in Heart Failure
AF78: 1-(3-Fluoro-4-(3-fluoropropoxy)phenethyl)guanidine
AFBG: (3-Astato-4-fluorobenzyl)guanidine
ASV: Adaptive servo-ventilation
BAT: Brown adipose tissue
BBB: Blood–brain barrier
BP_ND_: Nondisplaceable binding potential
C6-hNET: Rat C6 glioma cells stably transfected with the human NET
CHF: Congestive heart failure
CNS: Central nervous system
COMT: Catechol-*O*-methyl transferase
DMI: Desipramine
EDTA: Ethylenediaminetetraacetic acid
FDG: Fludeoxyglucose
FIBG: 4-Fluoro-3-iodobenzylguanidine
FMeNER-D2: 2-((S)-(2-((fluoro)methoxy-d2)phenoxy)(phenyl)methyl)morpholine
FPOIBG: 4-Fluoropropoxy-3-iodobenzylguanidine
GMO: *N*-Guanyl-meta-octopamine
H/B: Heart-to-blood
H/L: Heart-to-liver
HED: Hydroxyephedrine
HF: Heart failure
HFpEF: Heart failure with preserved left ventricular ejection fraction
HIBG: 4-Hydroxy-3-iodobenzylguanidine
ICD: Implantation of/implantable cardioverter defibrillators
LMI1195: *N*-[3-Bromo-4-(3-fluoro-propoxy)-benzyl]guanidine
LV: Left ventricular
MA1: Multilinear analysis 1
MAO: Monoamine oxidase
Me@HAPTHI: (S)-1-(3-hydroxy-4-(methylamino)butyl)-3-phenyl-1,3-dihydrobenzo[c][1,2,5]thiadiazole 2,2- dioxide
MBF: Myocardial blood flow
MDD: Major depressive disorder
MFBG: *meta*-Fluorobenzylguanidine
MHPG: 4-Fluoro-*m*-hydroxyphenethylguanidine
MIBG: *meta*-Iodobenzylguanidine
MRB: (S,S)-*O*-methyl-reboxetine
NE: Norepinephrine
NET: Norepinephrine transporter
NHP: Nonhuman primate
NS12137: Exo-3-[(6-fluoro-2-pyridyl)oxy]-8-azabicyclo[3.2.1]-octane
OCTs: Organic cation transporters
PCC: Pheochromocytoma
PD: Parkinson’s disease
PET: Positron emission tomography
PFBG: *para*-Fluorobenzylguanidine
PGL: Paraganglioma
PhB: Phenoxybenzamine
PHPG: 3-Fluoro-*p*-hydroxyphenethylguanidine
RI: Retention index
ROIs: Regions of interest
SERT: Serotonin transporter, also 5-HTT
SNS: Sympathetic nervous system
SPECT: Single-photon emission computed tomography
SUV_max_: Maximum standardized uptake value
VAMT2: Vesicular monoamine transporter 2
*V*_T_: Volume of distribution
WAT: White adipose tissue
